# A preliminary study on efficacy of rupatadine for the treatment of acute dengue infection

**DOI:** 10.1038/s41598-018-22285-x

**Published:** 2018-03-01

**Authors:** Gathsaurie Neelika Malavige, Ananda Wijewickrama, Samitha Fernando, Chandima Jeewandara, Anushka Ginneliya, Supun Samarasekara, Praveen Madushanka, Chameera Punchihewa, Shiran Paranavitane, Damayanthi Idampitiya, Chandanie Wanigatunga, Harsha Dissanayake, Shamini Prathapan, Laksiri Gomes, Siti A. B. Aman, Ashley St. John, Graham S. Ogg

**Affiliations:** 1 Centre for Dengue Research, Faculty of Medical Sciences, University of Sri Jayawardenapura, Jayawardenapura, Sri Lanka; 2 National Institute of Infectious Diseases, Angoda, Sri Lanka; 3 Program in Emerging Infectious Diseases, Duke-NUS Medical School, Singapore, Singapore; 4 Department of Pathology, Duke University, Duke, USA; 5 Department of Microbiology and Immunology, Yong Loo Lin School of Medicine, National University of Singapore, Singapore, Singapore; 6 MRC Human Immunology Unit, Weatherall Institute of Molecular Medicine, University of Oxford, Oxford, OX3 9DS UK

## Abstract

Currently there are no specific treatments available for acute dengue infection. We considered that rupatadine, a platelet-activating factor receptor inhibitor, might modulate dengue-associated vascular leak. The effects of rupatadine were assessed *in vitro*, and in a dengue model, which showed that rupatadine significantly reduced endothelial permeability by dengue sera *in vitro,* and significantly inhibited the increased haematocrit in dengue-infected mice with dose-dependency. We conducted a randomised, placebo-controlled trial in 183 adult patients in Sri Lanka with acute dengue, which showed that rupatadine up to 40 mg daily appeared safe and well-tolerated with similar proportions of adverse events with rupatadine and placebo. Although the primary end-point of a significant reduction in fluid leakage (development of pleural effusions or ascites) was not met, post-hoc analyses revealed small but significant differences in several parameters on individual illness days - higher platelet counts and lower aspartate-aminotransferase levels on day 7 in the rupatadine group compared to the placebo group, and smaller effusions on day 8 in the subgroup of patients with pleural effusions. However, due to the small sample size and range of recruitment time, the potential beneficial effects of rupatadine require further evaluation in large studies focused on recruitment during the early febrile phase.

## Introduction

The incidence of dengue has risen 30-fold during the past 50 years and there is a steady increase in the countries reporting dengue infection^1^. The dengue virus is a flavivirus, belonging to the same family as the Zika virus. It is estimated that 390 million dengue infections occurred in 2010, resulting in approximately 96 million clinically apparent infections^2^. The estimated annual global cost associated with dengue is $8.9 billion^3^. Currently there is no specific treatment for this infection and careful monitoring and administration of fluid remains the only treatment option^1^. There is clearly a major unmet clinical need for a safe effective therapy that can be realistically used in a practical way in resource-poor communities where the bulk of infections occur.

Vascular leakage is a hallmark of dengue haemorrhagic fever (DHF) and is thought to occur due to endothelial dysfunction^4^ resulting in increased vascular permeability. Severe dengue is characterized by clinically detectable vascular leak, which manifests clinically in the form of pleural effusions or ascites and can lead to haemodynamic instability and shock^1^. Occurrence of shock has been shown to be a key association with fatalities in dengue, followed by organ dysfunction and severe bleeding^5,6^. Although there are many potential causes of organ dysfunction and severe bleeding^7^, poor organ perfusion and poor blood supply to the intestinal mucosa have been suggested as one of the main causes of these complications^1^. Therefore, drugs that prevent or reduce vascular leakage, would be a suitable option to reduce severe dengue and associated complications. We recently reported that platelet activating factor (PAF) is elevated during acute dengue infection and may be an important mediator of vascular leak^8^. We found that sera from patients with acute dengue significantly reduced the expression of tight junction protein ZO-1, and also reduced trans-endothelial resistance (TEER) in endothelial cells, which were both significantly improved by PAF receptor blockade^8^. PAF has also been shown to be associated with vascular leak in mouse models of dengue infection^9^. PAF is rapidly synthesized from many cells such as endothelial cells, leucocytes, mast cells, macrophages and monocytes^10^. PAF is a potent inducer of increased vascular permeability in sepsis and anaphylaxis^11^ partly by promoting inter-endothelial leakage^12^. Overall these data support approaches to investigate the clinical effects of PAF receptor blockade in human dengue infection.

Rupatadine is an orally available second-generation antihistamine known to have long acting dual histamine-1-receptor blocking activities and PAF receptor blocking activities for treatment of allergic disease and chronic urticaria^13,14^. Rupatadine, which competitively blocks both histamine and PAF receptors, has been shown to be well tolerated in many clinical trials in patients who were treated for allergic rhinitis^13–15^, and off-label doses of up to 40 mg/day have shown to be well tolerated in chronic urticaria^16^. Rupatadine has not shown cardiac adverse effects even at administration of 100 mg in European and Asian individuals^17–19^. Given the PAF receptor blocking activity, we considered that rupatadine may be repurposed for use during acute dengue infection. It has advantages that it is administered orally and is relatively cheap and so could offer a realistic practical benefit in resource-poor clinical settings. We sought to test this possibility *in vitro*, in an animal model, and in a placebo controlled clinical trial.

## Results

### Effects of rupatadine on reducing endothelial cell permeability *in vitro*

**Figure 1:**
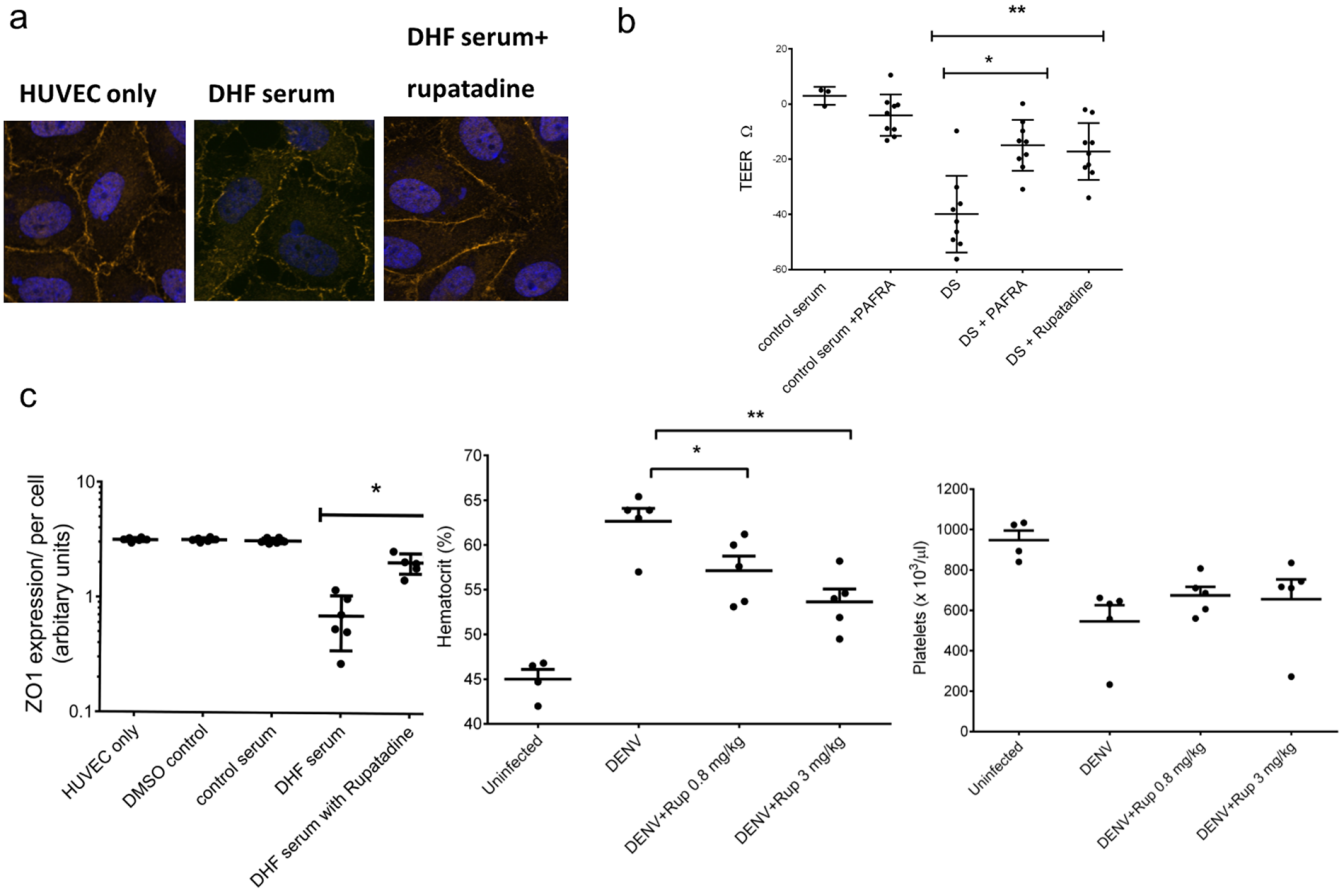
Rupatadine influences vascular function *in vitro* and in a dengue mouse model. (**a**) Human Umbilical vein endothelial cells (HUVEC) were stained for ZO-1 (red) and nuclei (DAPI, blue) incubated for 1 hour in the absence or presence of rupatadine (500 ng/ml) before adding DMSO or acute dengue serum (diluted 1:3 with media). Example of a stain from 1 of n = 6, experiments done in triplicate. ZO-1 expression was quantified as previously described with cumulative data from n = 6 experiments. (**b**) Trans-endothelial electrical resistance (TEER) of HUVEC cells was measured after incubation with acute dengue sera in the presence or absence of a PAFR antagonist (1-(N,N-Dimethylcarbamoyl)-4-ethynyl-3-(3-fluoro-4-((1H-2-methylimidazo[4,5-c]pyridin-1-yl)methyl)benzoyl)-indole, HCl) and rupatadine 500 ng/ml for 1 hour. n = 6 experiments done in triplicate. (**c**) Hematocrit values and platelet counts were obtained by automatic hematology analysis, 24 h after C57BL/6 infection with DENV-2 (1 × 10^6^ pfu, intraperitoneal) and 23.5 h after treatment with rupatadine at either 0.8 mg/kg or 3 mg/kg doses (or saline control injection). The data represent experiments on 5 mice in each group. Bars represent the mean and SEM *P < 0.05, **P < 0.01, ***P < 0.001, ****P < 0.0001.

We have previously shown that PAF receptor blockade inhibited the effects of acute dengue sera on endothelial permeability *in vitro* using a high affinity inhibitor (1-(N,N-Dimethylcarbamoyl)-4-ethynyl-3-(3-fluoro-4-((1H-2-methylimidazo[4,5-c]pyridin-1-yl)methyl)benzoyl)-indole, HCl)^8^. In order to investigate whether a licensed human medication with known PAF receptor blockade activity could also affect dengue sera-induced vascular permeability, human umbilical vein derived endothelial cell lines (HUVEC) were incubated with PAF and/or acute dengue sera in the presence or absence of rupatadine. Rupatadine 500 ng/ml significantly reduced PAF-induced effects on expression of the tight junction protein-ZO-1 (zonula occludens-1) and trans-endothelial electrical resistance (TEER) (Fig. 1). In the presence of acute dengue serum from patients with dengue haemorrhagic fever (DHF), the ZO-1 HUVEC surface staining becomes punctate, but with control serum or in the presence of rupatadine, the surface staining is linear. Furthermore, we observed that rupatadine significantly inhibited the effects of acute dengue sera on HUVEC expression of ZO-1 and TEER (Fig. 1). The effects of rupatadine on TEER were found to be comparable to that of the high affinity PAF receptor antagonist, 1-(N,N-Dimethylcarbamoyl)-4-ethynyl-3-(3-fluoro-4-((1H-2-methylimidazo[4,5-c]pyridin-1-yl)methyl)benzoyl)-indole, HCl. However, the concentration of rupatadine used in these *in vitro* experiments were higher than observed in humans, when given rupatadine 40 mg daily (Cmax = 15 ng/ml)^19^. These data confirmed that rupatadine could modulate the effects of PAF and acute dengue sera on human endothelia, raising the possibility that it may have clinical benefit during acute dengue infection.

### Effectiveness of rupatadine in reducing vascular leak in a dengue serotype 2 mouse model

Immunocompetent C57BL/6 mice have previously been used to evaluate the degree of vascular leak and rise in haematocrit following dengue infection^20,21^. Rupatadine was found to significantly reduce vascular leak when administered in a DENV-2 mouse model when used at 0.8 and 3 mg/kg, with evidence of dose-dependency (Fig. 1). The haematocrit did not return to baseline with rupatadine suggesting that PAF receptor blockade was incomplete, or the effects of the presence of other known mediators of vascular leak^22–25^. Although there was a trend towards inhibiting the dengue-associated thrombocytopenia following rupatadine treatment, this was not statistically significant. There were no increased bleeding events in the rupatadine treated mice. Taken with the *in vitro* data, these findings suggested that rupatadine indeed could reduce vascular leakage, possibly through PAF receptor blocking activity. Although it is likely that PAF is not the only mediator of vascular leak, the effects may be sufficient to impact clinical disease in humans, and we undertook a placebo controlled clinical trial.

### Pragmatic randomised placebo-controlled phase II study of rupatadine in dengue infection

In order to maximise data and to ensure patient safety, we designed an investigator-led, phase II, randomised, placebo-controlled study, consisting of three arms, in which two rupatadine doses (10 mg and 40 mg) were initially compared to placebo, but with the possibility of discontinuing one treatment arm should there be no evidence of benefit. We reasoned that doses above the licensed 10 mg rupatadine may be required and recruited individuals with acute dengue infection presenting sequentially to the National Institute of Infectious Diseases (NIID), of Colombo, Sri Lanka for up to 5 days (or until hospital discharge). As safety was a key aspect to the study, the patients and all medical personel who were caring for the patients were blinded, whereas the investigators who were recruiting the patients and recording the data from the patients’ clinical records were not blinded. The two primary objectives in this study, were to evaluate the safety of rupatadine up to 40 mg/day in acute dengue infection and its effect in preventing or reducing fluid leak. Accordingly, the primary efficacy endpoint assessed in the trial was defined as development of either ascites or pleural effusion at any time during follow up. Although a rapid rise in the haematocrit along with thrombocytopenia, is also considered as objective evidence of plasma leakage, as the haematocrit may also change due to dehydration or overhydration, we considered the detection of ascites or a pleural effusion as more objective evidence of plasma leakage. In our cohort, as all patients who developed pleural effusions also invariably had ascites, for analysis of the primary end point, the presence of any ascites was used as an indicator of fluid leakage. We hypothesised that rupatadine would be useful if it reduced plasma leakage from the expected level of 35% to 15% (a 20% difference). As three arms (*k* = 3) of administration were to be studied and the expected baseline response rate was 15% (*p* = 0.15) with 80 (*n* = 80) patients per arm, we have probability 0.99 (*Prob* = 0.99) of selecting the best treatment method which would reduce plasma leakage rate at 35% (*D* = 0.20)^26^.

The secondary objectives of this study were to assess reduction in complications including liver failure, reduction in the proportion of individuals who developed shock, reduction in the need of the use of colloids, reduction in the need of blood transfusions and a reduction in the duration of the illness. All patients aged between 18–60 years, with confirmed dengue infection (by a positive dengue NS1 antigen detection test) who gave informed written consent and who had a duration of illness ≤5 days and who did not show any evidence of vascular leak (pleural effusions or ascites) were recruited. The randomization for rupatadine 10 mg, 40 mg or the placebo was done in 1:1:1 ratio using a computerized random number generator of sequential patients with dengue attending the hospital.

#### Baseline characteristics

After recruitment of 38 individuals to the 10 mg rupatadine arm, there was no significant evidence of benefit of rupatadine over placebo, and so recruitment to this arm was stopped (Supplementary material), and subsequent patients were recruited to the placebo and 40 mg rupatadine arms only. The baseline characteristics were similar in those recruited for the rupatadine 40 mg arm and those recruited to the placebo arm (Table 1).

**Table 1:** Baseline characteristics of the patients at time of recruitment.

	Rupatadine 40 mg N = 66	Placebo N = 67
Age (mean, ± SD)	34.3 (11.2)	33 (11.3)
Sex		
Male (%)	53 (80.3)	51 (76.1)
Female (%)	13 (19.6)	16 (23.9)
Duration of illness at time of recruitment		
Median (IQR)	4 (3 to 4)	4 (3 to 4)
Mean (SD±)	3.7 (0.7)	3.7 (0.8)
≤3 days	17 (25.7)	21 (31.3)
>3 days	49 (74.2)	46 (68.6)
Infecting DENV serotype		
DENV1 (%)	44 (66.6)	46 (68.6)
DENV2 (%)	6 (9.1)	3 (4.5)
DENV3 (%)	0 (0)	0 (0)
DENV4 (%)	7 (10.6)	9 (13.4)
Negative (%)	9 (13.6)	9 (13.4)
Viral loads (pfu/ml) Median (IQR)	2006 (15.93 to 45, 243)	2722 (24.14 to 23,418)
Immune status		
Primary (%)	18 (27.3)	14 (20.9)
Secondary (%)	42 (63.6)	47 (70.1)
Inconclusive (%)	6 (9.1)	6 (8.9)
Haematocrit (median, IQR)	42 (39 to 44.8)	42 (38 to 44)
Platelet count (median, IQR)	112 (92.5 to 131.3)	110 (92 to 135)
AST (median, IQR)	76 (38.1 to 123.7)	75.9 (53.8 to 145.1)
ALT (median, IQR)	62 (29.7 to 110.1)	66.2 (35.8 to 104)

**Figure 2:**
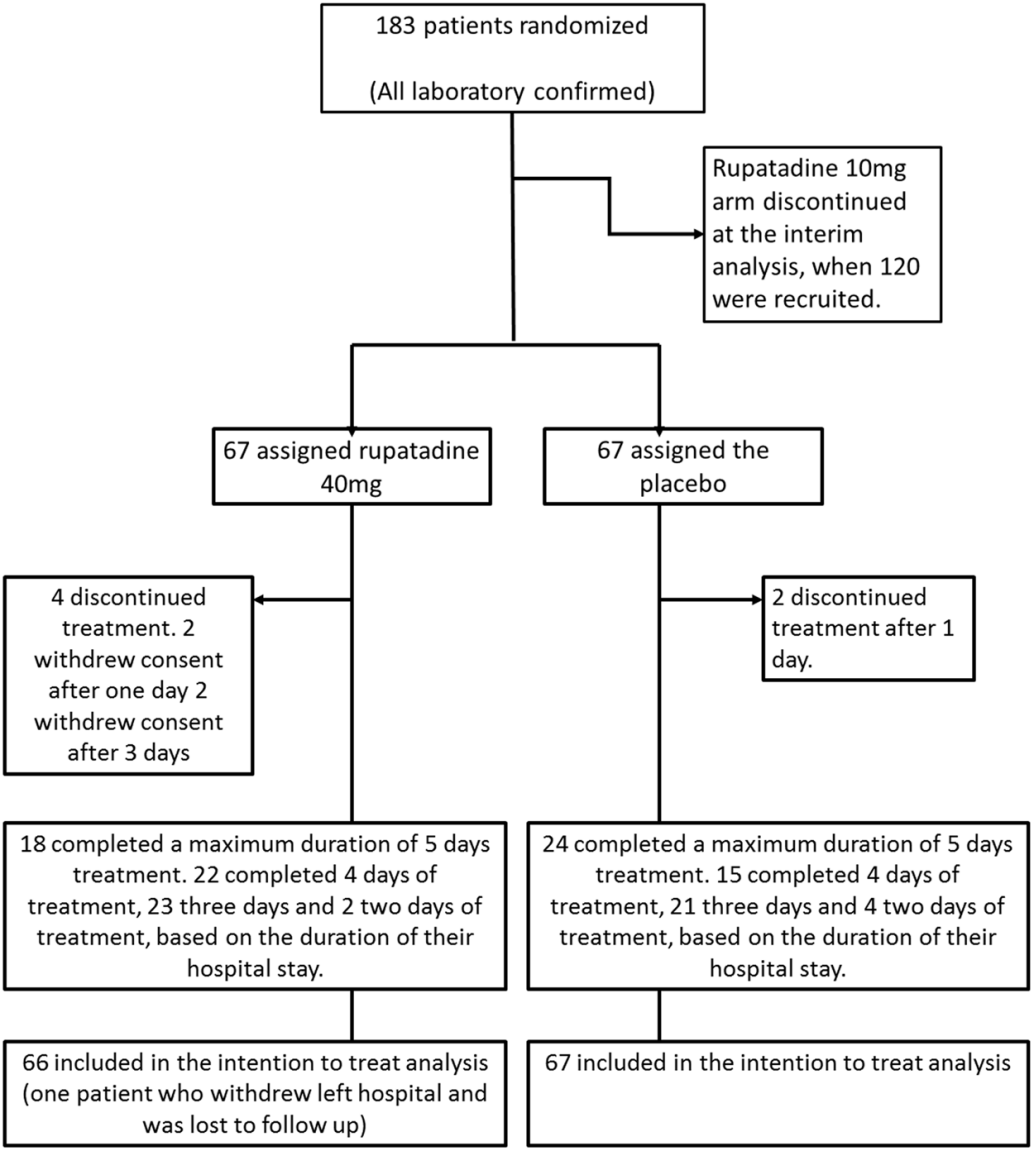
Overview of selection of randomization and evaluation of participants.

18 (27.3%) patients completed a maximum duration of 5 days of treatment in the rupatadine 40 mg arm and 24 (35.8%) in the placebo arm (Fig. 2). The duration of treatment was less for some individuals as they were discharged from hospital earlier because they had recovered and had no reason to be in hospital. The median duration of fever prior to presentation at the NIID was 4 days. By the time of presentation, most individuals had evidence of thrombocytopenia and liver dysfunction. DENV-1 was the dominant infecting serotype (90 patients, 67.7%), but there were also cases of DENV-2 (9 patients, 6.8%) and DENV-4 (16 patients, 12%). The baseline viral loads were similar in both arms at the time of recruitment. 32 (24.1%) of individuals had a probable primary infection and 89 (66.9%) had a probable secondary infection as determined by IgG and IgM status.

#### Adverse events

All clinical and laboratory adverse events (AE) were classified by using the Common Terminology Criteria for adverse events, which has been used in reporting of AE in previous clinical trials in acute dengue^27^. The number of adverse events (AE) were similar in those on rupatadine 40 mg and on the placebo and all AE completely resolved (Table 2). Two patients (2.98%) in the placebo group developed a SAE, namely acute liver failure. AE such as hepatitis (ALT >250U/L) were more frequent in those on the placebo as 6 (8.9%) patients developed hepatitis when compared to 3 (4.5%) on rupatadine 40 mg. Severe thrombocytopenia (<5 × 10^9^/L) was seen in one patient in the placebo group. Three of the four patients who withdrew from the 40 mg rupatadine arm and the two who withdrew consent from the placebo arm, did so as they did not wish additional blood being taken for the study. One of the patients who withdrew from the rupatadine arm left hospital and was lost to follow up – analysis was undertaken using intention to treat. None of the patients developed shock and there was no mortality in any study participant.

**Table 2:** Adverse events experienced by patients on rupatadine 40 mg and placebo.

	Rupatadine 40 mg N = 66	Placebo N = 67	P value
Abdominal pain (%)	17 (25.7)	21 (31.3)	0.5
Vomiting (%)	21 (31.8)	21 (31.3)	1.0
Diarrhoea (%)	27 (40.9)	24 (35.8)	0.7
Hepatitis (%) (ALT > 250/L)	3 (4.5)	6 (8.9)	0.3
Low platelet counts			
<20 × 10^9^/L (%)	12 (18.2)	14 (20.9)	0.8
20 to 50 × 10^9^/L (%)	17 (25.7)	21 (31.3)	0.2
High ALT (>200 U/L) (%)	9 (13.6)	12 (17.9)	0.2
High AST (>200 U/L) (%)	14 (21.2)	18 (26.9)	0.6
Low white cell count (<1 × 10^9^/L) (%)	0 (0)	0 (0)	1.00

#### Overall efficacy

**Figure 3:**
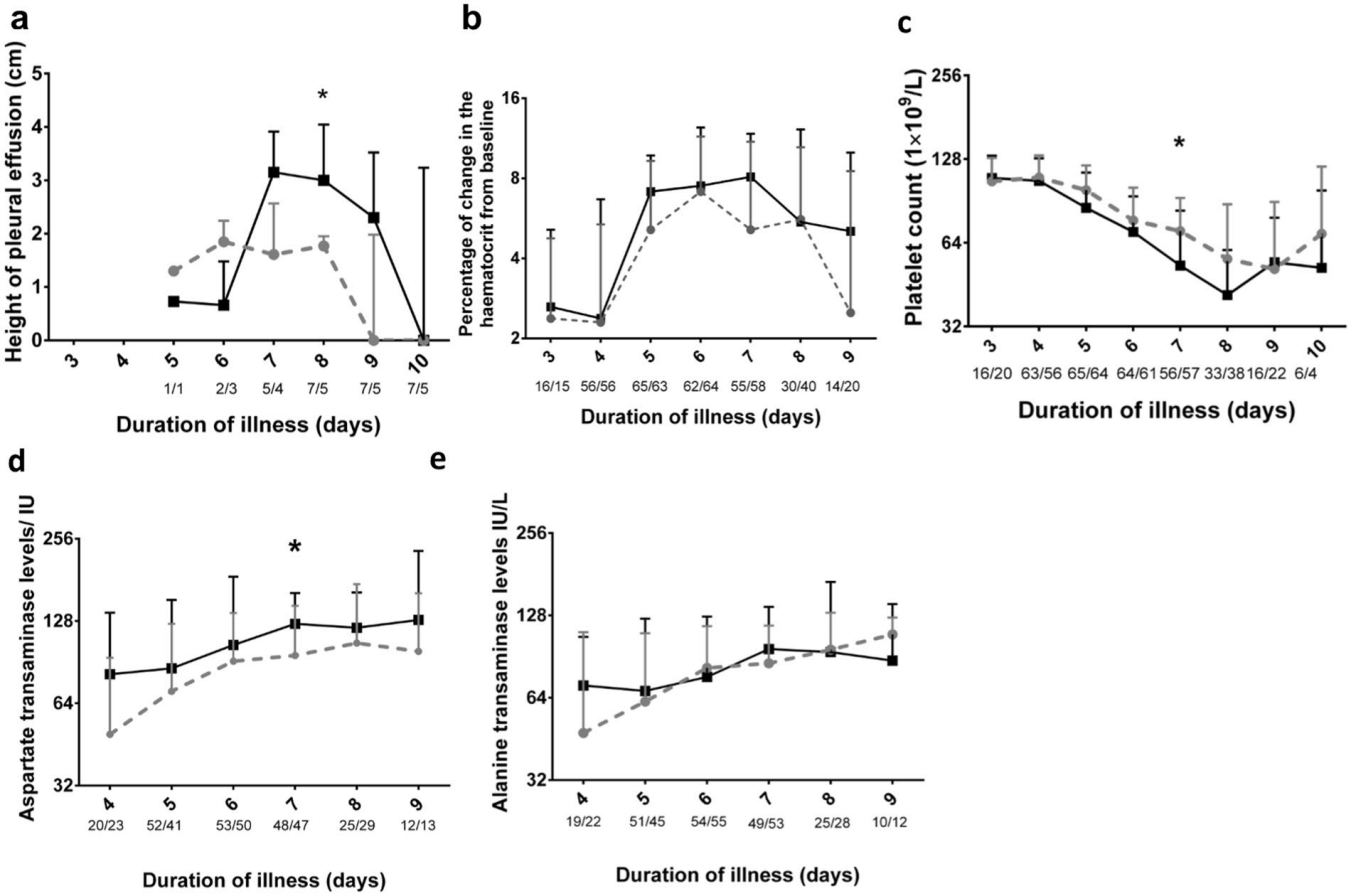
Changes in clinical and laboratory parameters in patients on 40 mg of rupatadine and placebo. (**a**) Height of the pleural effusion was measured by daily ultrasound scan and expressed in time (days) since onset of symptoms. Dotted line represents patients receiving rupatadine (40 mg daily, n = 66), and solid lines represent those receiving placebo (n = 67). (**b**) Haematocrit was measured daily and expressed as percentage change from the baseline value at presentation. Serum aspartate transaminase levels (**c**), alanine transaminase (**d**), and blood platelet counts were measured (**e**). The number of patients included for the analysis for each parameter, each day is indicated in the x-axis below each day (rupatadine/placebo). The bars represent median and the interquartile range *P < 0.05.

The primary efficacy endpoint (development of ascites or pleural effusions) was observed in similar numbers of participants in the two study arms. 15 (22.7%) in the rupatadine arm and 18 (26.9%) in the placebo arm, developed ascites, which was not significant by the Mann-Whitney test (p = 0.7) (Table 3). Among these patients, 7 patients in the rupatadine arm and 5 patients in the placebo arm developed pleural effusions, all of whom also had ascites. We found no difference in the median maximum height of these effusions when compared between the two study arms overall (Table 3), but when assessed by day of illness we observed a >50% reduction in the median of the maximum height of the pleural effusion on day 8 of illness (Fig. 3). By this time 5/7 patients in the rupatadine arm (1.7, range 0.0 to 2.1 cm) and 5/5 patients on the placebo arm (3.0, range 1.4 to 4.6 cm) had persisting pleural effusions, while the effusions in 2/7 patients in the rupatadine arm were no longer detectable. This was statistically significant when analysed using the Holm-Sidak method of calculation of p values correcting for multiple comparisons (p = 0.02). A rise in the haematocrit of >20% from the baseline, is also considered as evidence of plasma leakage according to the WHO guidelines^1^. All patients who developed a rise in the haematocrit of >20%, in this study, also had ascites. As the haematocrit can also change according to dehydration and overhydration, a rise of the haematocrit of >20% alone was not considered in the evaluation of the primary end point.

**Table 3:** Clinical outcomes of patients on 40 mg of rupatadine and on the placebo.

	40 mg rupatadine N = 66	Placebo N = 67	P value
Ascites by Ultra sound scan (%)	15 (22.7)	18 (26.9)	0.7
Extent of Ascites as measured by US Scan			
Mild (%)	5 (7.6)	8 (11.9)	0.56
Moderate (%)	10 (15.1)	10 (14.9)	0.7
Pleural effusion by Ultra Sound Scan (%)	7 (10.6)	5 (7.4)	0.8
Maximum height of pleural effusion			
Mean (SD±)	1.7 (0.4)	2.85 (1.3)	0.1
Haematocrit >20% rise from baseline	9 (13.6)	10 (14.9)	1.0
Acute liver failure (%)	0 (0)	2 (3.0)	0.24
Peak ALT levels (median, IQR)	102.3 (69.2 to 150)	106.9 (69 to 164.5)	0.7
Peak AST levels (median, IQR)	115.1 (76.8 to 181.5)	148 (88.2 to 213.5)	0.15
Duration of illness (days)			
Median (IQR)	7 (6 to 8)	8 (6 to 9)	0.09
Mean (SD±)	7.0 (1.4)	7.5 (1.5)	
Day of entering the critical phase (15 patients for rupatadine and 18 for placebo)			
Median (IQR)	5 (5 to 5)	4.5 (4 to 5)	**p** = **0**.**03**
Mean (SD±)	4.9 (0.9)	4.5 (0.8)	
Given normal saline boluses (%)	0 (0)	2 (2.98)	0.24
Given dextran (%)	6 (9.1)	6 (8.9)	1.0
Given blood (%)	0 (0)	1 (1.5)	0.5
Exceeded the fluid quota (%)	16 (24.2)	17 (25.4)	0.8
Quantity of extra fluid given Median (IQR)	362.5 (281.2 to 836.3)	575 (325 to 1000)	0.4
Platelet nadir during illness (median, IQR)	53.5 (29.7 to 85.5)	47 (24 to 83)	0.5
Bleeding (%)	2 (3.0)	6 (8.9)	0.3
Vaginal bleeding (% of females)	2 (15.4)	4 (25)	
Epistaxis	0 (0)	1 (1.5)	
GI bleeding	0 (0)	1(1.5)

In the planned analysis, we did not observe any overall differences in the secondary end points in the rupatadine or the placebo arm.

However, a significant reduction (p = 0.02) in AST levels were seen in patients on rupatadine (median 95.8, IQR 56.7 to 145.6 IU) on day 7 of illness compared to placebo (median 125.1, IQR 92.1 to 162.1). A significant reduction in thrombocytopenia (p = 0.01) was also seen in patients on rupatadine (median 70.5, IQR 41.25 to 92.75 cells/mm^3^) on day 7 of the illness compared to those on the placebo (median 53, IQR 29.5 to 83.5 cells/mm^3^) (Fig. 3). 6 (8.9%) patients on the placebo developed significant bleeding, which was considered as any bleeding that was not restricted to the skin. Four of these participants reported per vaginal bleeding when they were not menstruating (in 3 patients lasting for >3 days), one reported melena and one developed epistaxis. In contrast, 2 (3%) of patients in the rupatadine group developed per vaginal bleeding, which did not last for more than one day. There were no differences in other clinical and laboratory parameters, or the duration of illness in those on the placebo and rupatadine. As per national guidelines^28^, all patients underwent assessment several times a day to detect changes in the blood pressure, urine output, haematocrit and platelet counts to assess risk of fluid leakage and the onset of the critical phase. Those who develop fluid leakage (ascites or pleural effusions) as detected by ultra sound scans, are considered to have developed DHF and thus are considered to have entered the critical phase (fluid leakage phase). As 15 patients on rupatadine and 18 patients on the placebo developed some form of fluid leakage (pleural effusions or ascites), only these patients were considered to have entered the critical phase. We observed that all patients who developed pleural effusions, invariably had ascites. Interestingly, those who were on rupatadine entered the critical phase significantly later (p = 0.03) than those in the placebo (Table 3).

There were no differences in the clinical or laboratory outcomes in the treatment group or the placebo group in those with primary and secondary dengue infection, or with the different dengue serotypes.

#### Early efficacy of rupatadine

Although analysis of the effect of recruitment time was not a pre-specified analysis, we noted significant differences when rupatadine was administered at ≤3 days and felt that this was likely to be an important finding, worthy of post-hoc analysis, albeit in the knowledge that post-hoc analyses can risk bias. 17 (25.7%) patients who were assigned to the rupatadine arm and 21 (31.3%) patients assigned to the placebo arm were recruited at ≤3 days of illness. When rupatadine was given earlier, one of the primary end points was met, namely a >50% or more reduction in the fluid leakage, as assessed by the degree of ascites (mild or moderate). For instance, 4 (23.5%) in the rupatadine group developed ascites whereas 9 (42.8%) in the placebo group developed ascites, although this was not significant. Furthermore, as shown in Table 4, there was a reduction in the proportion of patients who developed pleural effusions (3 patients, 14.3% on placebo vs one patient, 5.9% on rupatadine), moderate ascites (4 patients, 19.4% vs one patient, 5.9%) and a >20% rise in the haematocrit (5 patients, 23.8% vs one patient, 5.9%). In addition, the proportion of patients who had AST levels of >200 U/L was also higher in the placebo group (6 patients 28.5% vs 1 patient, 5.9% given rupatadine). We did not find any differences in the reduction in viraemia in those who were recruited early (≤3 days) in the placebo or the rupatadine group (Supplementary Figure 1).

**Table 4:** Clinical and laboratory features of patients who were on rupatadine or on the placebo who were recruited on ≤ 3 days.

	Placebo Recruited ≤3 days N = 21	Rupatadine Recruited ≤3 days N = 17	P value
Ascites by Ultra sound scan (%)	9 (42.8)	4 (23.5)	0.3
Extent of Ascites as measured by US Scan (%)			
Mild	5 (23.8)	3 (17.6)	0.7
Moderate	4 (19.4)	1 (5.9)	0.3
Pleural effusion by Ultra Sound Scan (%)	3 (14.3)	1 (5.9)	0.6
Haematocrit >20% rise from baseline	5(23.8)	1 (5.9)	0.3
Duration of illness			
Median (IQR)	7 (6 to 8)	7 (5.5 to 7)	0.17
Mean (SD±)	7.1 (1.8)	6.3 (1.5)	
Given dextran (%)	3 (14.3)	1 (5.9)	0.6
Exceeded the fluid quota (%)	6 (28.6)	4 (23.5)	1.0
Quantity of extra fluid given Median (IQR)	362.5 (193.8 to 545)	300 (231.3 to 627.5)	0.4
Hepatitis (ALT >250/L) (%)	0 (0)	0 (0)	1.0
High ALT (>200 U/L) (%)	2 (9.5)	1 (5.9)	1.0
High AST (>200 U/L) (%)	6 (28.5)	1 (5.9)	0.1
Bleeding (%)	2 (9.5)	2 (11.7)	1.0
Low platelet counts			
<20 × 10^9^/L (%)	5 (23.8)	2 (11.7)	0.4
20 to 50 × 10^9^/L (%)	7 (33.3)	7 (41.2)	1.0

Within the group who were given rupatadine, the overall duration of illness was significantly less in those who were recruited early (early treatment group), when compared to those recruited after 3 days (p = 0.03). Of those who were on rupatadine and treated early, only one patient (5.9%), developed a pleural effusion, when compared to 6 (12.2%) of those who were recruited later than 3 days (Table 5), but this did not reach significance. Although the proportion of patients who developed ascites was similar, the severity was less in the early treated group in which only one patient (5.9%) developed moderate ascites compared to nine (18.4%) of those who were recruited late. Of those who were recruited early only one (5.9%) patient was given colloids (dextran) in anticipation of subsequent deterioration, when compared to 5 (10.2%) of those who were recruited later. Only one patient (5.9%) had a rise in the haematocrit >20% at some time during the when compared to 8 (16.3%) in the late treatment group. Although the lower frequency of complications observed in those who were recruited early, within the rupatadine group, could be attributed to earlier detection of complications and management, a lower frequency of complications was not observed in those within the placebo group, who were recruited early (Table 6). For instance, 3 (14.3%) of those who were recruited early (≤3 days) in the placebo group developed pleural effusion, compared to 2 (4.3%) of those who were recruited after 3 days. Nine (42.8%) who were recruited early developed ascites, compared to 9 (19.5%) recruited later and 3 (14.3%) who were recruited early, required dextran compared to 1 (5.9%) recruited later (Table 6). Therefore, although it appears that within the rupatadine treatment group, those who were recruited earlier (and therefore had two or more doses of rupatadine) had fewer complications compared to those on rupatadine recruited later, this was not observed within the placebo group. However, as sample numbers are small and this was a post-hoc analysis, larger studies are required for firm conclusions.

**Table 5:** Clinical and laboratory outcomes of patients who were given rupatadine 40 mg either ≤3 days of illness or later.

	Rupatadine 40 mg Recruited ≤3 days N = 17	Rupatadine 40 mg Recruited >3 days N = 49	P value
Ascites by Ultra sound scan (%)	4 (23.5)	11 (22.4)	1.0
Extent of Ascites as measured by US Scan			
Mild (%)	3 (17.6)	2 (4.1)	
Moderate (%)	1 (5.9)	9 (18.4)	0.4
Pleural effusion by Ultra Sound Scan (%)	1 (5.9)	6 (12.2)	0.7
Haematocrit >20% rise from baseline	1 (5.9)	8 (16.3)	0.4
Duration of illness			
Median (IQR)	7 (5.5 to 7)	7 (6.5 to 8)	
Mean (SD±)	6.3 (1.5)	7.3 (1.4)	**0**.**03**
Given dextran (%)	1 (5.9)	5 (10.2)	1.0
Exceeded the fluid quota (%)	4 (23.5)	12 (24.5)	1.0
Quantity of extra fluid given			
Median (IQR)	300 (231.3 to 627.5)	362 (281.3 to 812.5)	0.4
Hepatitis (ALT > 250/L) (%)	0 (0)	3 (6.1)	0.56
High ALT (>200 U/L) (%)	1 (5.9)	8 (16.3)	0.4
High AST (>200 U/L) (%)	1 (5.9)	13 (26.5)	0.09
Bleeding (%)	2 (11.7)	0 (0)	0.06
Low platelet counts			
<20 × 10^9^/L (%)	2 (11.7)	10 (20.4)	0.7
20 to 50 × 10^9^/L (%)	7 (41.2)	10 (20.4)	0.4

**Table 6:** Clinical and laboratory outcomes of patients who were given the placebo either ≤3 days of illness or later.

	Placebo Recruited ≤3 days N = 21	Placebo Recruited >3 days N = 46	P value
Ascites by Ultra sound scan (%)	9 (42.8)	9 (19.5)	0.1
Extent of Ascites as measured by US Scan			
Mild (%)	5 (23.8)	3 (6.7)	0.09
Moderate (%)	4 (19.4)	6 (13.3)	0.07
Pleural effusion by Ultra Sound Scan (%)	3 (14.3)	2 (4.3)	0.3
Haematocrit > 20% rise from baseline	5 (23.8)	5 (10.9)	0.3
Duration of illness			
Median (IQR)	7 (6 to 8.5)	8 (6.5 to 9)	0.3
Mean (SD ± )	7.1 (1.8)	7.6 (1.3)	
Given dextran (%)	3 (14.3)	1 (5.9)	0.1
Exceeded the fluid quota (%)	6 (28.6)	4 (23.5)	0.1
Quantity of extra fluid given			
Median (IQR)	362.5 (193.8 to 545)	300 (231.3 to 627.5)	0.08
Hepatitis (ALT > 250/L) (%)	0 (0)	6 (13.3)	0.2
High ALT (>200 U/L) (%)	2 (9.5)	10 (21.5)	1.0
High AST (>200 U/L) (%)	6 (28.5)	12 (26.5)	1.0
Bleeding (%)	2 (9.5)	4 (8.7)	1.0
Low platelet counts			
<20 × 10^9^/L (%)	5 (23.8)	9 (20)	0.75
20 to 50 × 10^9^/L (%)	7 (33.3)	14 (31.1)	0.61

## Discussion

PAF was previously found to be an important contributor to vascular leak and PAF receptor blockade was found to inhibit the effects of acute dengue sera on the expression of the tight junction protein ZO-1, and in the reduction of trans-endothelial resistance^8^. Rupatadine is an orally available, and relatively inexpensive therapeutic that is known to have PAF receptor blockade activity. Indeed, rupatadine significantly blocked the effects of acute dengue sera on the expression of ZO-1 and TEER by human endothelial cells. The effect of rupatadine *in vivo* was confirmed in an immunocompetent dengue serotype 2 mouse model, where it significantly reduced the rise in haematocrit in dengue infected mice. Collectively, these data suggested that repurposing rupatadine might be effective in reducing vascular leakage in acute dengue infection.

Rupatadine is a second generation antihistamine as well as having a PAF receptor blockade activity, and has been used for treatment of allergic rhino-conjunctivitis^29^, chronic urticaria^30^, cold urticaria^31,32^ and mastocytosis^33^. Many clinical guidelines recommend using up to four times the standard dose of rupatadine (40 mg) in the treatment of chronic urticaria if required^34,35^, which was found to be well tolerated^19^. In this phase II clinical trial, we found that rupatadine appeared safe and well tolerated at 10 mg and 40 mg a day in patients with acute dengue infection. The AE experienced by those on rupatadine were similar to those on the placebo. Although the trial was initially started with 3 arms, the 10 mg arm was discontinued as it showed no benefit over the placebo, while the 40 mg rupatadine appeared to show some benefit at the interim analysis. The study was conducted among adults, and 71.4% of patients recruited had been ill for at least 4 days. Therefore, most only received one dose of the drug before entering the critical phase, which was typically on day 5 of illness. Although the proportion of those who developed vascular leak (those who developed ascites or pleural effusions) was similar in those on the 40 mg arm and the placebo arm, when assessed by day of illness, we observed a >50% reduction in the median of the maximum height of the pleural effusion on day 8. Interestingly, the time taken to enter the critical phase (fluid leakage phase) was significantly longer in those on rupatadine 40 mg, which could be due to the reduction in the fluid leakage. For instance, fluid leakage was evaluated by detecting either pleural effusions or ascites, and even though ultra sound scans were used for their detection, small amounts of fluid could go undetected. Although not significant, the post-hoc analysis showed that the occurrence of pleural effusions, ascites and requirement for extra colloids such as dextran, were less in the patients recruited early (≤3 days of illness) to the rupatadine arm when compared to the placebo arm. The analysis carried out within the rupatadine arm also showed a reduction in the frequency of complications in those who were recruited early (and therefore were given more doses of rupatadine) when compared to those were recruited later, although this was not significant. However, such differences were not seen within the placebo group when comparing those who were recruited earlier vs those who were recruited later. Therefore, although it is possible that those who were given more than one dose of rupatadine (as they were recruited earlier) before developing fluid leakage had fewer complications, it is difficult to draw firm conclusions, due to the small sample size included in the post-hoc analysis.

PAF is released from platelets and many other cells, and has roles in platelet aggregation, and so it is noteworthy that the frequency of haemorrhage was lower in the rupatadine 40 mg group compared to placebo. However, since rupatadine also blocks histamine receptors, it would be important to determine whether the effects of rupatadine where due to the blocking of the PAF receptor alone, or whether its antihistamine effects had any benefit, for example through future studies of pure PAFR blockade. Rupatadine has also been shown to inhibit degranulation of mast cells in animal models and inhibit the release of leukotrienes^36^. Since mediators released from mast cells have been shown to contribute to vascular leak in dengue mouse models and humans^20,21,37,38^, rupatadine could possibly further contribute to reduction in complications due these additional effects.

Many drug trials have been conducted previously to determine efficacy in acute dengue infection^27,39–43^. Many of these trials have evaluated the efficacy of antivirals^40–43^, while some have investigated immunomodulatory drugs such as steroids^39^. Among the antiviral drugs, chloroquine has shown to significantly reduce the duration of fever and showed a trend towards reduction in the proportion of patients developing DHF^41^. Celgosivir was found to be safe and showed a trend towards reduction in the platelet nadir and trend towards reduction in a rise in the haematocrit in patients with secondary dengue^42,43^. In a randomised, placebo controlled trial consisting of a high dose and low dose treatment regimens of steroids, the investigators did not find any differences in the virological or clinical endpoints in those in the high dose or low dose treatment arm compared to the placebo^39^. Although these trials assessed the proportion of patients who developed fluid leakage (DHF), they did not carry out ultra sound examinations daily, to quantify the fluid leakage by assessing the development of ascites or pleural effusions. The main reason we quantified daily fluid leakage in our trial was because even if a drug fails to completely prevent fluid leakage, a significant reduction in the amount of leakage will significantly reduce the burden associated with acute dengue infections. Fatalities due to dengue occur due to shock followed by organ dysfunction and severe bleeding, which are complications of severe vascular leakage^1,5,6^. Therefore, even though a drug may not completely prevent vascular leakage, if a drug can significantly reduce leakage, it would be helpful in reducing fatalities due to dengue.

Limitations of the study included the single blind trial design which was used to enable more rapid detection of safety signals given the known role of PAF in haemostasis, but reasonable attempts were made to minimise bias, such as having distinct teams for recruitment, care and analysis. In addition, the multiple comparisons performed for this study would have limited the power of the statistical analysis. It has been shown that rupatadine was less effective in individuals of Chinese origin due to CYP3A polymorphisms^44^. Therefore, future trials should incorporate pharmacokinetic assessments. The earliest we could recruit patients was at day 3 of fever, which may miss greater benefits in patients treated earlier still. The advent of bedside NS1 testing in community practice may facilitate earlier diagnosis and recruitment in future trials. Doses above 40 mg/day rupatadine were not assessed, and these may offer further therapeutic benefit. The study was not powered to determine effects on mortality (no deaths in any arm as dengue associated mortality rates are <0.02% in Sri Lanka), which would be important to address in future larger trials. Overall, we are encouraged by the use of rupatadine, particularly in early dengue infection, but larger multi-center trials will undoubtedly be important before any changes to clinical practice are advised.

In conclusion, rupatadine was shown to inhibit effects of PAF and acute dengue sera on HUVEC cells *in vitro* and to inhibit fluid leakage in a DENV-2 model *in vivo*. Rupatadine 40 mg appeared to be safe and well tolerated in patients with acute dengue. We found no direct evidence of benefit in the main endpoints evaluated, but post-hoc analysis revealed small but significant differences in several parameters on individual illness days. A larger clinical trial focused on early enrolment is needed to clarify the significance of these potential signals of efficacy.

## Materials and Methods

### Determining effect of rupatadine on human endothelial cell lines *in vitro*

Human umbilical vein derived endothelial cell lines (HUVEC) were used to determine the effects of rupatadine at a concentration of 500 ng/ml in reducing PAF-induced and dengue patient sera-induced effects (n = 6) on expression of tight junction protein-ZO-1 and its effects on trans-endothelial electrical resistance (TEER). The dengue patient sera were from patients with DHF, who had severe vascular leak (shock). Ethics approval was obtained from the Ethics Review Committee of University of Sri Jayewardenapura and informed written consent was obtained from all patients. The clinical disease severity was classified according to the 2011 WHO dengue diagnostic criteria^1^ and accordingly shock was defined as having cold clammy skin, along with a narrowing of pulse pressure of ≤20 mmHg. All experiments were done in triplicate and five image fields per condition were obtained to include in the analysis. Culture, maintenance of HUVECs and determining ZO-1 expression and trans-endothelial resistance was carried out as previously described^8^.

### Determining the effect of rupatadine in dengue 2 mouse model

C57BL/6 mice were infected with Eden2, a clinical isolate of DENV serotype 2 and the effects of rupatadine in reducing vascular leak and on thromobocytopenia were assessed as previously described^20,21^. C57BL/6 wild type mice were used instead of IFNα receptor of STAT-2 knockout mice as we expected the wild type mice as a better reproduction of pathology, albeit recognising that no mouse model reflects perfect recapitulation. Briefly, the infection was initiated by injection of 1 × 10^6^ pfu of a DENV-2, intraperitoneally. Rupatadine 0.8 mg/kg and 3 mg/kg or saline as a control were also injected intraperitoneally 30 minutes after infection. Vascular leak was assessed by measuring the haematocrit after 24 hours of infection as previously described, by an automatic hematology analyzer^21^. All animal experiments were performed according to protocols approved by the SingHealth Institutional Animal Care and Use Committee.

### Patient recruitment and data collection

Patients admitted to a dedicated dengue management unit in a tertiary care hospital in Colombo District, Sri Lanka (National Infectious Diseases Hospital), were recruited following informed written consent.

### Human Protocol Methods

The trial was approved by the Ethics Review Committee of the University of Sri Jayewardenapura and also the SCOCT of the Ministry of Health, Sri Lanka. The trial was registered at the Sri Lanka Clinical trial registry on the 2^nd^ of October 2014 (International Clinical Trials Registration Platform: SLCTR/2014/023) and the trial recruited patients from January 2015 to July 2016. All methods involving human patients were performed in accordance with the relevant guidelines and regulations.

#### Inclusion and exclusion criteria

All patients aged between 18–60 years, with confirmed dengue infection (by a positive dengue NS1 antigen detection test) who gave informed written consent and who had a duration of illness ≤5 days and who did not show any evidence of vascular leak were recruited (a baseline ultra sound scan was performed to exclude any patients with ascites or pleural effusions).

Although we hypothesised that giving the drug early during the illness would probably be of benefit, in Sri Lanka and many other dengue affected countries, adult patients are usually only admitted to hospital on day 4 of illness or later. Therefore, since this clinical trial was carried out in a hospital setting, we recruited patients up to day 5 of illness. All pregnant women, those who report reactions to antihistamines or relevant excipients, those who are alcohol dependent or abused drugs or those with previously diagnosed hepatic or renal impairment were also excluded. Those who were using alcohol and narcotics were excluded due to the possible derangements that these substances might have caused at the time of recruitment and therefore, would influence the liver transaminase levels and possible development of acute liver failure.

### Trial design

The clinical trial was an investigator-led phase II, randomized controlled study, initially consisting of three arms, which were rupatadine 10 mg, rupatadine 40 mg and placebo. As safety was a key aspect to the study, and since it is recommended that rupatadine should be used with caution in patients with liver dysfunction, which is a known complication of acute dengue^7^, and due to its potential effects on platelet aggregation in the setting of the thrombocytopenia and since some patients would be on four times the licensed dose of the drug (in allergy) a single-blinded design was used. However, in order to collect unbiased data, the investigators who recruited patients and administered the drug were completely separate from the group of doctors who managed the patients. The medical personnel managing the patients recorded the clinical data, and decided on fluid treatment and when to discharge from hospital and had no knowledge of which drug the patients were given. The investigators who were involved in recruiting and administering drugs, recorded all clinical data from the hospital records and so could detect safety signals early.

An interim analysis was carried out after recruiting half of the number of patients (n = 120). At the interim analysis, it was found that 40 mg rupatadine was safe in acute dengue, and that 10 mg of rupatadine did not seem to show any benefit when compared to the placebo, and so the rupatadine 10 mg arm was discontinued (supplementary data). 63 further patients were recruited following informed written consent for the remaining two arms. Details regarding the trial protocol are available as Supplementary Appendix 1. We have adhered to the CONSORT guidelines for publication when analysing and reporting our data^45^. An independent drug safety monitoring board (DSMB), which consisted of an experienced biostatistician, a clinical pharmacologist and two professors of medicine in two other universities, assessed the safety outcomes, overall study integrity and also advised on protocol amendments. The 10 mg rupatadine arm was discontinued on their advice.

### Randomization and masking

The randomization for rupatadine 10 mg, 40 mg or the placebo was done in 1:1:1 ratio using a computerized random number generator of sequential patients with dengue, admitted to the hospital. After discontinuation of the 10 mg arm, randomization was done in a 1:1 ratio (Fig. 2). Once the patient was recruited, he/she was given a unique code. The patients who received the 40 mg dose received 4 tablets of 10 mg rupatadine tablets daily for a maximum of 5 days from the day of recruitment. The control group received 4 placebo tablets daily for a maximum of 5 days from the day of recruitment. Those on 10 mg of rupatadine received one tablet of 10 mg rupatadine and three tablets of the placebo. The drug was given for a maximum of 5 days as patients with acute dengue are unlikely to be hospitalized for more than 5 days unless they develop significant complications and once admitted to hospital, they are likely to fully recover within 5 days since admission.

The patients received the drugs once the randomization was completed and they were assigned to a study arm and the drugs were administered each morning. The rupatadine tablets were provided by the pharmaceutical manufacturers Dr. Reddy in India, and Sri Lanka Pharmaceutical Manufacturing Corporation provided the placebo which was the same size, shape and colour. They played no part in the study design, analysis or reporting. Participants were kept blind to the treatment allocation for the duration of study. All groups received the standard supportive care treatment as per national guidelines with no other differences between groups^28^.

### Study procedures

The patients were monitored clinically throughout their stay in hospital by the staff of the ward (several times a day) and were only discharged from hospital once the physicians taking care of the patients, decided that they were fit to be discharged. Up to 5 days since the day of recruitment (median day 4 since onset of illness) was felt to be an appropriate length of follow-up as this is standard medical practice in Sri Lanka and we wished the study findings to be valid for such a setting. The patients had completely recovered at the time of discharge (median day 9 since onset of illness). At the point of discharge, no further data were obtained from the research participants, and no carry forward of existing data was used. The haematocrit and platelet counts were assessed several times a day and the liver transaminases and ultrasound scans were done daily. Details of assessment of the extent of fluid leakage (presence of ascites and pleural effusions) are described in Supplementary Appendix 1. The group of investigators who recruited the patients (those who were not involved in their management) recorded all clinical and laboratory data of these patients, which were entered in the hospital records by the ward staff.

Dengue was confirmed by detection of virus by quantitative multiplex, real time PCR^7^ and by detection of dengue specific IgM and IgG antibodies by ELISA (Panbio, Australia) as previously described. The dengue IgM and IgG antibody testing was performed on the blood sample taken on the last day at hospital, which was after day 7 of illness. Patients who only had dengue virus specific IgM were classified as having a primary dengue infection while those who had a positive result for both IgM and IgG were classified as having a secondary dengue infection^46^. Those who had an equivocal result shown for dengue IgM and IgG by ELISA according to the manufacturers’ instructions, in the blood sample taken at the time of discharge from hospital were categorised as having an inconclusive serological status.

Details of clinical disease severity such as development of DHF, bleeding and acute liver failure and adverse events were recorded and reported to the Ethics Review Committee. Serious adverse events were reported to the Ethics Review Committee and to the independent DSMB.

### Objectives and outcomes

There were two primary objectives in this study, which were to evaluate the safety of rupatadine up to 40 mg/day in acute dengue infection and its effect in preventing or reducing fluid leak. In order to determine if rupatadine prevented fluid leakage, we assessed if there was a reduction in the proportion of individuals who developed fluid leakage (those who developed pleural effusions or ascites) in the treatment arm compared to the placebo. However, as all patients who had pleural effusions, invariably also had some degree of ascites, the presence of ascites was used for analysis of fluid leakage, when evaluating the primary end point. Although a rise in the haematocrit of >20% of the baseline value, along with a drop in the platelet count is also considered as objective evidence of fluid leakage, we did not consider the changes in the haematocrit for the analysis of the extent of fluid leakage, as the haematocrit varies with dehydration and overhydration. As we also wished to assess if rupatadine, reduced the extent of leakage, the quantity of pleural effusion or ascites, were measured in whom fluid was detected in pleural or peritoneal cavities. For evaluation of reduction in the pleural fluid leakage, the maximum height of the pleural effusion was assessed daily in all patients, throughout the course of the illness. The maximal distance between mid-height of the diaphragm and visceral pleura was measured in millimeters. We assessed the median of the maximum height of the pleural effusion observed in those on the placebo and evaluated if there was more than 50% reduction in the median of the maximum height of the pleural effusion observed during the illness, in those who were on rupatadine. The degree of pleural effusion is a known measure used to assess dengue disease severity^47–49^. The quantity of ascites was assessed by semi-quantitatively by looking for the presence of fluid in five areas of the abdomen namely RUQ (perihepatic and Morrison’s pouch), LUQ (perisplenic), right paracolic gutter, left paracolic gutter and pelvis. Specific primary endpoints were a reduction in the proportion of individuals who were treated with rupatadine who develop fluid leakage; and reduction in fluid leakage by 50% or more in those who were treated with rupatadine. As all patients who had pleural effusions, invariably also had some degree of ascites, the presence of ascites was used for analysis of fluid leakage, when evaluating the primary end point.

The secondary objectives of this study were to assess reduction in complications including liver failure, reduction in the proportion of individuals who developed shock, reduction in the need of the use of colloids, reduction in the need of blood transfusions and a reduction in the duration of the illness. Details of definitions used for acute liver failure, shock and duration of illness are given in Supplementary information. The secondary outcomes were analysed by assessing the differences in the proportion of individuals on rupatadine vs placebo who developed liver failure, shock, the proportion who needed less colloids than the amount allocated initially and the need for blood transfusions. The reduction in the duration of illness was assessed by the differences in the mean duration of illness in those on the placebo compared to rupatadine. The first day of illness was defined as the day in which the patient developed fever and the day of recovery was defined as the patient being afebrile for 24 hours, the platelet counts rising to >50,000 cells/mm^3^ or a rise of 20% from the lowest platelet value, and the return of the haematocrit to the patient’s baseline. The duration of illness was taken as the number of days between the day of onset of illness and recovery.

### Statistics

We hypothesized that if rupatadine was to be tested at phase II level, it would only be considered useful in the management of dengue and worth undertaking a phase III trial, if it reduced plasma leakage (presence of pleural effusions or ascites) from the expected level of 35% to 15% (a 20% difference). As three arms (*k* = 3) of administration were to be studied and the expected baseline response rate was 15% (*p* = 0.15) with 80 (*n* = 80) patients per arm, we have probability 0.99 (*Prob* = 0.99) of selecting the best treatment method which would reduce plasma leakage rate at 35% (*D* = 0.20)^26^. Therefore, 80 patients were to be selected in each arm. However, after the interim analysis as the 10 mg arm was discontinued, the new sample size was calculated based on the interim data using a two-tailed, z-test of proportions between the two groups with 80% power and a 5% level of significance, based on the same reduction of plasma leakage percentages used in sample calculation in the initial 3 arms. According to this calculation, it was predicted that a sample size of 130 patients, 65 in each arm (rupatadine 40 mg and the placebo), was sufficient to detect a clinically important difference of 20% between groups in reducing plasma leakage.

Statistical analysis was performed using Graph PRISM version 6 and non-parametric statistical tests were used. Differences in the serial values of the height of the pleural effusions, liver enzymes, platelet counts and changes in haematocrit values in patients on the two arms of treatment were done using multiple unpaired non-parametric t tests. Corrections for multiple comparisons were done using Holm-Sidak method and the statistical significant value was set at 0.05 (alpha). Longitudinal analyses were undertaken using 2-way repeated measures ANOVA. The significance of the differences in the duration of illness and the time at entering the critical phase were compared using the Mann-Whitney t test (two tailed).

### Data availability

All data are available in the manuscript and supporting files.

## Electronic supplementary material

Supplementary data
