# Supplementary material for: A preliminary study on efficacy of rupatadine for the treatment of acute dengue infection
Source: Sci Rep. 2018 Mar 1;8:3857. doi: 10.1038/s41598-018-22285-x (PMC5832788; doi:10.1038/s41598-018-22285-x)
Supplement: Supplementary file 1 — Supplementary data [file 41598_2018_22285_MOESM1_ESM.docx]

**A preliminary study on efficacy of rupatadine for the treatment of acute dengue infection**

Gathsaurie Neelika Malavige^1,6*^, Ananda Wijewickrama^2^, Samitha Fernando^1^, Chandima Jeewandara^1^, Anushka Ginneliya^1^, Supun Samarasekara^1^, Praveen Madushanka^1^, Chameera Punchihewa^1^, Shiran Paranavitane^1^, Damayanthi Idampitiya^2^, Chandanie Wanigatunga^1^, Harsha Dissanayake^1^, Shamini Prathapan^1^, Laksiri Gomes^1^, Siti A.B. Aman^3^, Ashley St. John^3,4,5^, Graham S. Ogg^1,6^

Centre for Dengue Research, Faculty of Medical Sciences, University of Sri Jayawardenapura, Sri Lanka^1^; National Institute of Infectious Diseases, Angoda, Sri Lanka^2^; Program in Emerging Infectious Diseases, Duke-NUS Medical School, Singapore^3^; Department of Pathology, Duke University, USA; Department of Microbiology and Immunology^4^, Yong Loo Lin School of Medicine, National University of Singapore^5^; MRC Human Immunology Unit, Weatherall Institute of Molecular Medicine, University of Oxford, OX3 9DS UK^6^

**Primary objectives**

The two primary objectives were to evaluate the effect of rupatadine in preventing or reducing fluid leakage and safety in patients with acute dengue. Specific primary endpoints were a reduction in the proportion of individuals who were treated with rupatadine who develop fluid leakage (pleural effusions or ascites); and reduction in fluid leakage by 50% (reduction of ascites or the maximum height of the pleural effusions) or more in those who were treated with rupatadine. However, as all patients who had pleural effusions, invariably also had some degree of ascites, the presence of ascites was used for analysis of fluid leakage, when evaluating the primary end point.

Rupatadine was to be considered safe in patients with acute dengue if there were no statistically significant differences in the changes in biochemical and hematological parameters between the 3 arms apart from those defined as outcomes and those that are expected in patients with dengue. We also evaluated any other patient volunteered adverse effects and compare with existing literature on rupatadine.

**Secondary objectives**

The secondary objectives of this study are to evaluate reduction in complications associated with acute dengue including liver failure, reduction in the proportion of individuals who develop of shock, reduction in the need of the use of colloids, reduction in the need of blood transfusions and a reduction in the duration of the illness.

**Definitions**

Evidence of plasma leakage: evidence of plasma leakage was considered as detection of free fluid in the abdomen or by the presence of a pleural effusion by Ultra sound scan or by the presence of a rise in haematocrit of >20% of the baseline (*1*). Although a rise in the haematocrit of >20% of the baseline value, along with a drop in the platelet count is also considered as objective evidence of fluid leakage, according to the WHO 2011 guidelines (*1*), we did not consider the changes in the haematocrit for the analysis of the extent of fluid leakage, as the haematocrit varies with dehydration and overhydration. We only considered the occurrence of pleural effusions or ascites, which can be objectively evaluated by ultrasound scans as objective evidence of plasma leakage. However, in this study, as all patients with pleural effusions had ascites and since only those with ascites (along with pleural effusions) had a rise in the haematocrit of >20%, presence of ascites was used in evaluating the primary end point.

The quantity of fluid leakage was assessed by a semi quantitative measure, with classification of ascites as minimal, mild, moderate, severe and massive by looking for the presence of fluid in five areas of the abdomen namely RUQ (perihepatic and Morrison's pouch), LUQ (perisplenic), right paracolic gutter, left paracolic gutter and pelvis. For pleural effusion, the maximal distance between mid-height of the diaphragm and visceral pleura was measured in millimeters.

All individuals (3 individuals) who performed the ultra sound scans were trained by a specialist radiologist who is a member of the team of investigators. The images of all ultra sound scans performed by these 3 individuals, were saved and reviewed individually by the specialist radiologist. In addition, throughout the trial, random visits were made by the radiologist to ensure the same technique was used.

Liver failure: Liver failure was diagnosed based on published criteria, with all of the 3 following criteria present in a patient with a rise in liver transaminase levels (*2, 3*), namely the absence of any previously known liver disease, a prolonged prothrombin time of 4-6 seconds or more and an INR of >1.5.

Shock: Shock was defined as a pulse pressure of ≤20 mmHg or a drop in the systolic blood pressure of 30mmHg or more.

Duration of the illness: The first day of the illness was defined as the day in which the patient developed fever. The day of recovery was defined when all of the following three criteria are fulfilled:

- Patient afebrile for 24 hours
- Platelet counts of >50,000 cells/mm^3^ or a rise of 20% from the lowest value
- Return of the haematocrit to the patient’s baseline

Entering the critical phase: As serial ultrasound scans were carried out daily from the day of recruitment to discharge, the day in which pleural effusions or ascites was first detected was taken as the day the patient entered the critical phase.

Calculation of the percentage change in the haematocrit

The full blood counts were performed at least three times a day since the day of admission to hospital and the full blood counts were performed more frequently in the critical phase (4 to 6 times a day) as per National Dengue Management Guidelines. The haematocrit at the time of admission was considered as the baseline haematocrit, as all patients who were recruited were confirmed to not have fluid leakage at the time of recruitment. The percentage rise in the haematocrit was calculated as the percentage change in the haematocrit from the baseline to the maximum rise in the haematocrit as indicated in the WHO dengue guidelines.

Calculation of excess amount of fluid given

Excess fluid was the amount of fluid that given above the standard protocol of administering maintenance requirements plus 5% deficit, during the critical phase as per National Dengue Management Guidelines.

**Randomization procedure and drug dispensation**

The randomization for rupatadine 10mg, 40mg or the placebo was be carried out in 1:1:1 ratio using a computerized random number generator. The three treatments were named A (10mg rupatadine), B (40mg of rupatadine) and C (placebo). Once the patient was recruited, he/she was given a unique code. The patients who received a 10mg dose received 3 tablets of placebo in addition to the tablet of 10mg of rupatadine on a daily basis. The control group received 4 placebo tablets daily. Patients in the 40mg test group were given a daily dose of four 10mg rupatadine tablets orally starting on the day of recruitment. The patients received treatment for a maximum period of 5 days. All groups also received the standard supportive care treatment as per national guidelines and there were no other treatment differences (*4*). If the patient recovered before 5 days (from the day they were recruited to the study), the drug was stopped on the day of recovery. The patients were monitored throughout their stay in hospital and were only discharged from hospital, once the physicians taking care of the patients, decided that they were fit to be discharged. The patients were not followed up after discharge as they had fully recovered when they left hospital and complications due to acute dengue are rarely observed in patients with acute dengue who had recovered fully from the acute illness.

Investigators in this clinical trial who are medically qualified were to be recruiting patients, obtaining consent and answering any questions regarding the study. They themselves administered the drug, recorded the daily clinical features of the patients from the hospital records, and also carried out US scans. The investigators who were involved in recruitment and placebo/active administration were different from to those who were involved in the management of the patients. Investigators who managed the patients and recorded their the clinical data in hospital records and took decisions regarding hospital discharge, were not informed of the type of drug or dose received by the patients.

**Sample size**

We hypothesized that rupatadine was to be tested at phase II and it would only be considered useful in the management of dengue and worth undertaking a phase III trial, if it reduced plasma leakage (occurrence of ascites or pleural effusions) from the expected level of 15-20%. As three arms (*k*=3) of administration were to be studied and the expected baseline response rate was 15% (*p*=0.15) with 80 (*n*=80) patients per arm, we have probability 0.99 (*Prob*=0.99) of selecting the best treatment method which would reduce plasma leakage rate at 35% (*D*=0.20) (*5*). Therefore, 80 patients were to be selected in each arm. At the interim analysis, the efficacy of the two rupatadine arms was to be evaluated, and if there was no evidence of differences with placebo then an arm would be stopped at the interim stage (*6*).

However, after the interim analysis as the 10mg arm was discontinued, the new sample size calculation was done as follows. It was predicted that a sample size of 130 patients, 65 in each arm (rupatadine 40mg and the placebo), was sufficient to detect a clinically important difference of 20% between groups in reducing plasma leakage (occurrence of pleural effusions or ascites in these patients). This was calculated by using a two-tailed, z-test of proportions between the two groups with 80% power and a 5% level of significance.

Clinical evaluation

The patients were monitored twice a day (or more if required) by one of the study physicians throughout the duration of hospital stay as for all other patients admitted to this unit. All clinical features and laboratory investigations were recorded twice a day including fluid requirements, blood transfusions and all drugs that were administered. The fluid requirement for each patient was assessed individually if the patient developed fluid leakage (ascites or pleural effusions). Ultrasound Scans were done daily from the time of admission until the time of discharge from the hospital.

Laboratory evaluation

Full blood counts were done twice or more daily as required in the management of the patients. Liver function tests were done daily when blood samples were collected for the study. Dengue was confirmed by detection of virus by quantitative real time PCR or by detection of dengue specific IgM and IgG antibodies by ELISA.

**Safety reviews and the role of the data and safety monitoring board (DSMB)**

An independent data safety monitoring board was set up and assessed the safety of the drug once 50% (120 patients) of the targeted sample size was recruited. Analysis was by intention to treat. The data safety monitoring board consisted of university academics and researchers who were competent in conducting clinical trials and who had experience in treating patients with dengue. None of the investigators were present in the DSMB.

**Ethical Considerations**

Ethics Approval: Ethics approval was granted by the Ethics Review Committee, Faculty of Medical Sciences, University of Sri Jayawardenapura and also the Drug Regulatory Authority, Ministry of Health, Sri Lanka. The trial was registered with the Sri Lanka Clinical Trials Registry, and the WHO clinical trials registry: SLCTR/2014/023.

Informed consent and information sheet: The patients were given the patient information sheets of the study and were given an opportunity to discuss their concerns/questions with a member of the team of investigators. Once the patient was satisfied with the information given to him/her, he/she was given the opportunity to join the study or to decline. They were made aware that their decision will in no way affect their treatment and they were free to withdraw at any time.

**Supplementary results**

**Summary of the Trial data presented to the DSMB**

1. Number of total patients recruited: 120

2. Number of patients given 40mg of rupatadine: 40 (one withdrew on day 2)

3. Number of patients given 10mg: 36 (2 withdrew on day 2)

4. Number of patients given the placebo: 40 (one withdrew)

|  | Rupatadine 40mg  N=39 | Rupatadine 10mg  N=38 | Placebo  N=39 |
| --- | --- | --- | --- |
| Ascites by Ultra sound scan | 7 | 8 | 8 |
| Extent of Ascites as measured by US Scan  Mild  Moderate | 2  5 | 2  6 | 3  5 |
| Pleural effusion by Ultra Sound Scan | 4 | 6 | 4 |
| Acute liver failure | 0 | 0 | 2 |
| Peak ALT levels (median, IQR) | 94.7 (49.1 to 134) | 147.4 (83.2 to 202.7) | 98.6 (66.2 to 179) |
| Peak AST levels (median, IQR) | 109.8 (66.5 to 190.3) | 127.3 (77.8 to 205.8) | 127.9 (78.7 to 186.1) |
| Duration of illness (days)  Median (IQR) | 7 (6 to 8 days) | 7 (7 to 9 days) | 8 (6.5 to 9 days) |
| Number who exceeded allocated fluid quota | 8 | 9 | 8 |
| Number who required less than the fluid quota | 3 | 2 | 0 |
| Given dextran | 4 | 5 | 5 |
| Platelet nadir during illness (median, IQR) | 65 (41.5 to 86) | 53 (24 to 72.75) | 48 (26 to 78) |
| Bleeding | 0 | 3 (2 melena and 1 PV bleeding) | 4 (2 PV bleeding, one melena, one epistaxis) |
| Abdominal pain | 11 | 9 | 11 |
| Hepatomegaly | 12 | 11 | 10 |
| Vomiting | 15 | 12 | 10 |
| Diarrhoea | 17 | 19 | 19 |

**Table: Clinical outcomes of patients on 40mg, 10mg of rupatadine and on the placebo at the time of the interim analysis**

**References**

1. WHO, Ed., *Comprehensive guidelines for prevention and control of dengue fever and dengue haemorrhagic fever*, (World Health Organization, SEARO, New Delhi, India, 2011), vol. 60.

2. D. McDowell Torres, R. D. Stevens, A. Gurakar, Acute liver failure: a management challenge for the practicing gastroenterologist. *Gastroenterology & hepatology* **6**, 444-450 (2010).

3. T. Singh, N. Gupta, N. Alkhouri, W. D. Carey, I. A. Hanouneh, A guide to managing acute liver failure. *Cleve Clin J Med* **83**, 453-462 (2016).

4. S. L. Ministry of Health, in *National Guidelines*. (Ministry of Health, Sri Lanka, 2012).

5. R. Simon, R. E. Wittes, S. S. Ellenberg, Randomized phase II clinical trials. *Cancer treatment reports* **69**, 1375-1381 (1985).

6. C. Jeewandara *et al.*, Platelet activating factor contributes to vascular leak in acute dengue infection. *PLoS neglected tropical diseases* **9**, e0003459 (2015).





**Supplementary Fig 1**: Changes in viral loads in patients who were recruited early (≤3 days of illness) in patients who were on rupatadine (n=17, dotted line) and the placebo (n=21, solid line). The bars represent mean and SEM. There was no difference in the viral loads in those on rupatadine 40mg or the placebo.
